# The prognostic value of node status in different breast cancer subtypes

**DOI:** 10.18632/oncotarget.13943

**Published:** 2016-12-15

**Authors:** Zheng-Jun Yang, Yue Yu, Xin-Wei Hou, Jiang-Rui Chi, Jie Ge, Xin Wang, Xu-Chen Cao

**Affiliations:** ^1^ The First Department of Breast Cancer, Tianjin Medical University Cancer Institute and Hospital, National Clinical Research Center for Cancer, Tianjin 300060, China; ^2^ Key Laboratory of Cancer Prevention and Therapy, Tianjin 300060, China; ^3^ Key Laboratory of Breast Cancer Prevention and Therapy, Tianjin Medical University, Ministry of Education, Tianjin 300060, China

**Keywords:** breast cancer, nodal metastases, prognosis, molecular subtype

## Abstract

Nodal metastases and breast cancer subtypes (BCS) are both well-recognized prognostic indicators. However, the association between nodal metastases and BCS, and the prognostic value of nodal metastases in different BCS are still remains unclear. Our aim was to investigate the association between nodal metastases and BCS, and the prognostic value of nodal metastases in the different BCS.

We found that the breast cancer subtype was closely associated with the pN stage. pN stage and breast cancer subtype were significantly associated with disease-free survival. The subgroup analysis showed that the patients in higher pN stage had a poor outcome than patients in lower pN stage in each breast cancer subtype. Furthermore, when the analysis was stratified by breast cancer subtype, we found that even in the same pN stage (pN0-pN2), there was significant survival difference among patients in different BCS, and Luminal A breast cancer patients had the best survival outcome. However, there were no significant survival difference between Luminal A patients and other breast cancer subtype when patients in pN3 stage. Thus, our study suggested that both lymph node status and molecular subtype played important roles in the outcome of breast cancer patients and they cannot replace each other.

## INTRODUCTION

Breast cancer is one of the most commonly diagnosed cancers all over the world and a major cause of cancer-related death in women in China [1]. Lymph node metastasis is a well-known indicator of breast cancer metastases and associated with a poor survival outcome compared with patients with lymph node negative. Recent studies had shown that breast cancer subtypes are also associated with prognosis [2–8]. Although breast cancer subtypes were initially described and classified by molecular subtypes, immunohistochemical tumor assessment has been found to adequately estimate the molecular analysis with a simpler and more practical method for determining subtypes in the clinical setting [9, 10].

In addition, the 2011 St. Gallen International Expert Consensus Group has endorsed the use of IHC-based molecular subtypes as a surrogate for the intrinsic subtypes of breast cancer [11]. So, in now days, breast cancer subtypes are usually determined by using IHC surrogates and classified into four subtypes. A patient's tumor was considered triple negative (TNBC) if ER and PgR were both absent and HER2 status was negative; HER2-overexpression if ER and PgR were both absent and HER2 was positive; luminal A if ER- and/or PgR-positive, HER2-negative, and Ki-67 less than 14%; or luminal B if ER- and/or PgR-positive, HER2-negative, and Ki-67 ≥ 14% or if ER- and/or PgR-positive and HER-2 was positive. These four breast cancer subtypes are widely used in in the clinical setting to provide important insight into management strategies and risk of distant metastasis. Although nodal metastases and breast cancer subtypes are well-recognized prognostic indicators for breast cancer patients. However, whether there is an association between nodal metastases and breast cancer subtypes, and the prognostic value of nodal metastases in different breast cancer subtypes are still remains unclear. To our knowledge, we firstly investigated the association between nodal metastases and breast cancer subtypes with a large sample size.

At the same time, the prognostic value of nodal metastases in different breast cancer subtypes were also examined. Furthermore, after the analysis was stratified by breast cancer subtype, we found that although in the same pN stage (pN0-pN2), there were significant survival difference among patients in different breast cancer subtypes, and Luminal A breast cancer patients had the best survival outcome. However, there were no significant survival difference between Luminal A patients and other breast cancer subtype in patients with pN3 stage disease. Taken together, our results suggested that both lymph node metastases and molecular subtype played important role in affecting the survival outcome of breast cancer patients and they still seemed to be the most important indicators of prognosis.

## RESULTS

### Clinicopathological features and treatment modalities

**Figure 1:**
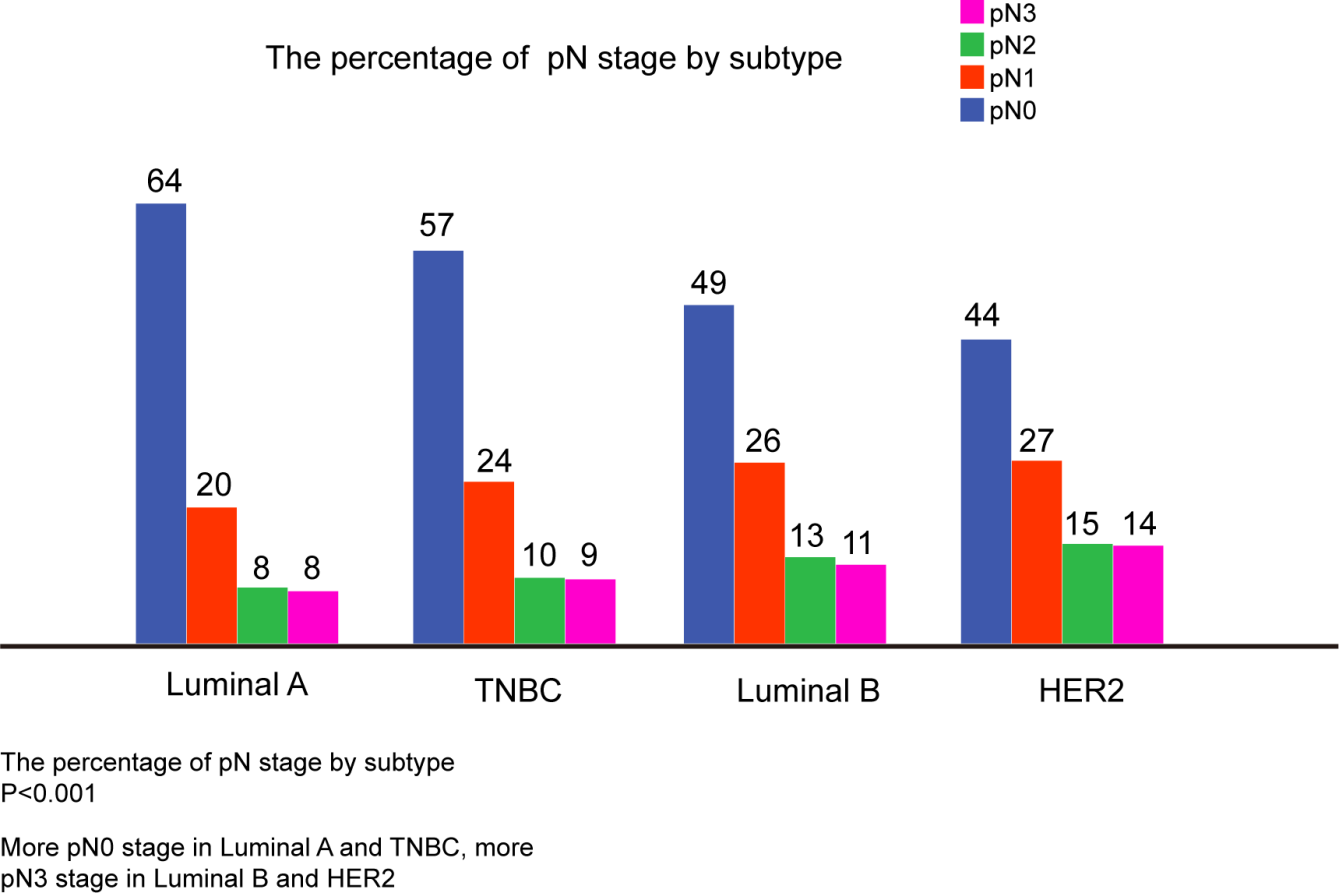
The percentage of Lymph Node Positivity by Subtype More pN0 in luminal A and TNBC, more pN3 in luminal B and HER2. TNBC Triple-negative breast cancer, vs. versus, HER2. HER2-overexpression.

A total of 4, 262 patients with invasive ductal carcinoma of the breast in our hospital were analyzed. Clinicopathological features and treatment modalities are summarized in Table 1. A total of 735 patients (17.2%) were Luminal A, 2, 324 patients (54.5%) were Luminal B, 825 patients (19.4%) were TNBC, and 378 (8.9%) patients were HER-2-overexpression. Of these 4, 262 patients, a total of 1, 997 patients (46.9%) were lymph node positive. There were significant associations with breast cancer subtype and age (*P* = 0.048), menopausal status (*P* = 0.014), pT stage (*P* < 0.001), pN stage (*P* < 0.001), histologic grade (*P* < 0.001) and soft tissue invasion (*P* < 0.001). However, there were no significant associations with breast cancer subtype and lymphatic invasion (*P* > 0.05). As shown in Figure 1, Luminal A and TNBC breast cancers were more frequently node-negative when compared to luminal B and HER2 cancers and less frequently in pN3 stage. All patients received modified radical mastectomies and no patients received neoadjuvant chemotherapy. The between subgroup test of interaction were shown in Supplementary Table S1A–S1L. After surgery, 3, 643 patients received adjuvant chemotherapy and 798 patients received adjuvant radiotherapy. Most of the patients with ER+/PR+ tumor received hormonal therapy at least 5 years, however, only few patients with Her2 positive breast cancer treated with Trastuzumab. Adjuvant chemotherapy regimens including cyclophosphamide+methotrexate+ fluorouracil (CMF), cyclophosphamide+adriamycin/epirubicin+fluorouracil (CAF/CEF), docetaxel+ adriamycin/epirubicin (TA/TE), and docetaxel+cisplatin (TP).

**Table 1:** Clinicopathological features and treatment modalities at presentation by breast cancer subtypes

Characteristic	Luminal A	Luminal B	TNBC	HER-2	*P* value
**Age**					**0.048**
<65	654	2084	765	341	
≥ 65	81	240	60	37	
**Menopausal status**					**0.023**
Premenopausal	417	1278	446	179	
Postmenopausal	318	1046	379	199	
**pT stage**					**< 0.001**
T1	217	750	236	80	
T2	486	1434	539	273	
T3	32	140	50	25	
**pN stage**					**< 0.001**
N0	473	1150	474	168	
N1	146	614	195	101	
N2	60	293	79	55	
N3	56	267	77	54	
**Histologic Grade**					**< 0.001**
I	80	130	39	8	
II	620	1965	619	294	
III	35	229	167	76	
**Soft tissue invasion**					**< 0.001**
No	647	1919	727	311	
Yes	88	405	98	67	
**Lymphatic invasion**					0.420
No	721	2289	812	368	
Yes	14	35	13	10	
**Adjuvant chemotherapy**					**< 0.001**
No	131	331	105	52	
Yes	604	1993	720	326	
**Adjuvant radiotherapy**					**< 0.001**
No	629	1812	721	302	
Yes	106	512	104	76	

TNBC. triple-negative, HER-2. HER-2 overexpression.

*P* value < 0.05 was considered to be significant, and significant *P* value was in bold font.

### Outcomes, including recurrence, and survival

At the last time of follow-up, 3, 507 of 4, 262 (82.3%) patients were alive and disease free, 552 (12.9%) were alive with recurrent cancer, and 203 (4.8%) died of recurrent cancer. As shown in Table 2, pT stage (*P* < 0.001), pN stage (*P* < 0.001), histologic grade (*P <* 0.001), soft tissue invasion (*P <* 0.001), lymphatic invasion (*P* = 0.012), breast cancer subtype (*P <* 0.001), adjuvant chemotherapy (*P* = 0.024) were significant predictors for DFS in univariate analysis. When these variables were analyzed with Cox proportional hazard model, pT stage (*P* < 0.001), pN stage (*P* < 0.001), histologic grade (*P* = 0.004), breast cancer subtype (*P <* 0.001), and adjuvant chemotherapy (*P* = 0.001) remained significant independent predicators for DFS.

**Table 2:** Univariate and multivariate analysis of clinicopathological variables affecting DFS

Variables	DFS			
	Univariate analysis		Multivariate analysis	
	HR (95% CI)	*P* value	HR (95% CI)	*P* value
**Age**		0.083		
< 65	1			
≥ 65	1.231 (0.974–1.556)			
**Menopausal status**		0.068		
Premenopausal	1			
Postmenopausal	1.143 (0.990–1.319)			
**pT stage**		**< 0.001**		**< 0.001**
T1	1		1	
T2	1.848 (1.530–2.228)		1.406 (1.161–1.703)	
T3	5.173 (4.001–6.688)		2.270 (1.730–2.978)	
**pN stage**		**< 0.001**		**< 0.001**
N0	1		1	
N1	2.134 (1.749–2.603)		2.387 (1.951–2.920)	
N2	3.654 (2.941–4.540)		4.685 (3.716–5.905)	
N3	8.137 (6.714–9.861)		10.197 (5.220–12.649)	
**Histologic Grade**		**< 0.001**		0.084
I	1		1	
II	3.478 (2.084–5.804)		1.776 (1.235–2.555)	
III	4.323 (2.519–7.419)		2.461 (1.641–3.351)	
**Soft tissue invasion**		**< 0.001**		0.727
No	1		1	
Yes	2.727 (2.330–3.191)		0.966 (0.795–1.174)	
**Lymphatic invasion**		**< 0.001**		0.263
No	1		1	
Yes	3.138 (2.164–4.549)		1.245 (0.848–1.827)	
**Breast cancer subtype**		**< 0.001**		**< 0.001**
Luminal A	1		1	
Luminal B	2.012 (1.559–2.595)		1.669 (1.292–2.155)	
TNBC	2.302 (1.736–3.053)		2.107 (1.588–2.796)	
HER-2	3.876 (2.855–5.262)		2.850 (2.095–3.877)	
**Adjuvant chemotherapy**		**< 0.001**		**0.033**
No	1		1	
CMF	0.531 (0.370–0.762)		0.674 (0.450–0.989)	
Anthracycline	0.456 (0.305–0.681)		0.632 (0.440–0.908)	
Anthracycline+Taxane	0.369(0.252–0.541)		0.585 (0.404–0.848)	
Other	0.525(0.363–0.758)		0.655 (0.444–0.967)	
**Adjuvant radiotherapy**		**< 0.001**		**< 0.001**
No	1		1	
Yes	0.360 (0.289–0.448)		0.408 (0.337–0.494)	
**Anti-HER-2 therapy**		**0.040**		0.816
No	1		1	
Yes	0.555 (0.317–0.974)		0.816 (0.364–1.826)	

TNBC. triple-negative, HER-2. HER-2 overexpression.

*P* value < 0.05 was considered to be significant, and significant *P* value was in bold font.

**Figure 2:**
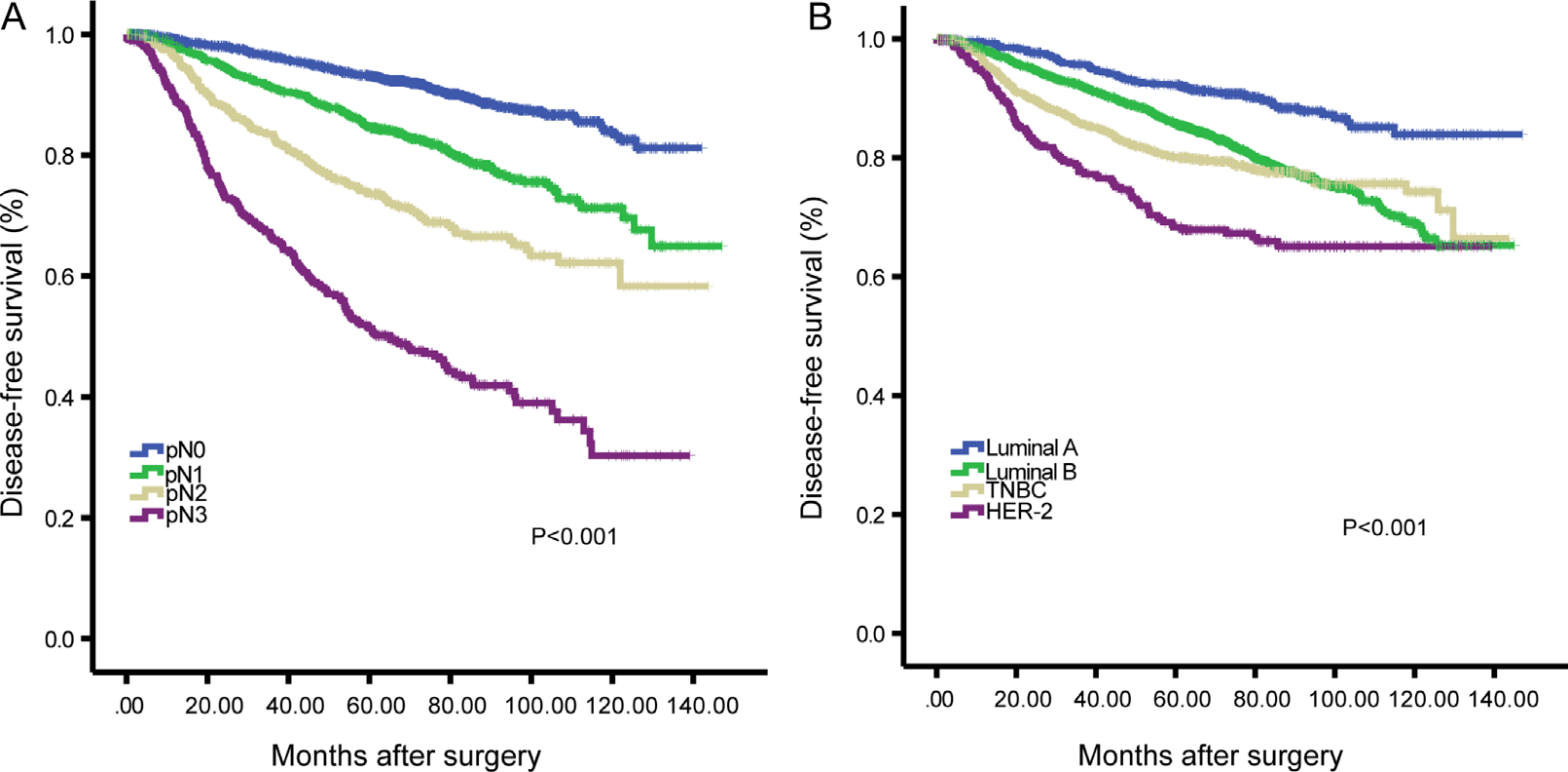
Kaplan-Meier analysis of the disease-free survival (DFS) according to the pN stage (**A**) and breast cancer subtypes (**B**).

**Figure 3:**
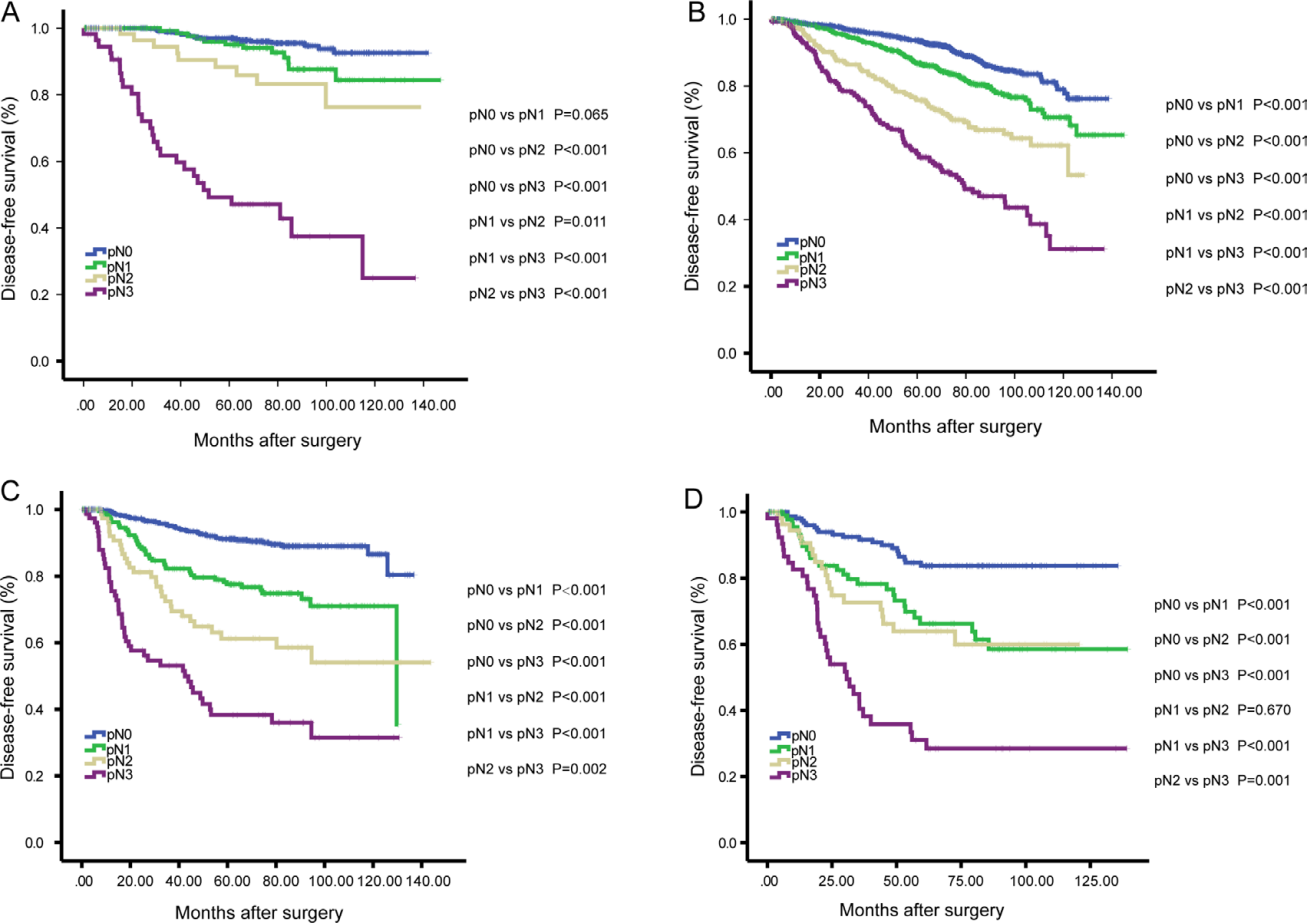
Kaplan-Meier analysis of the disease-free survival (DFS) according to the pN stage among patients with Luminal A (**A**), Luminal B (**B**), TNBC (**C**) and HER2 (**D**).

**Figure 4:**
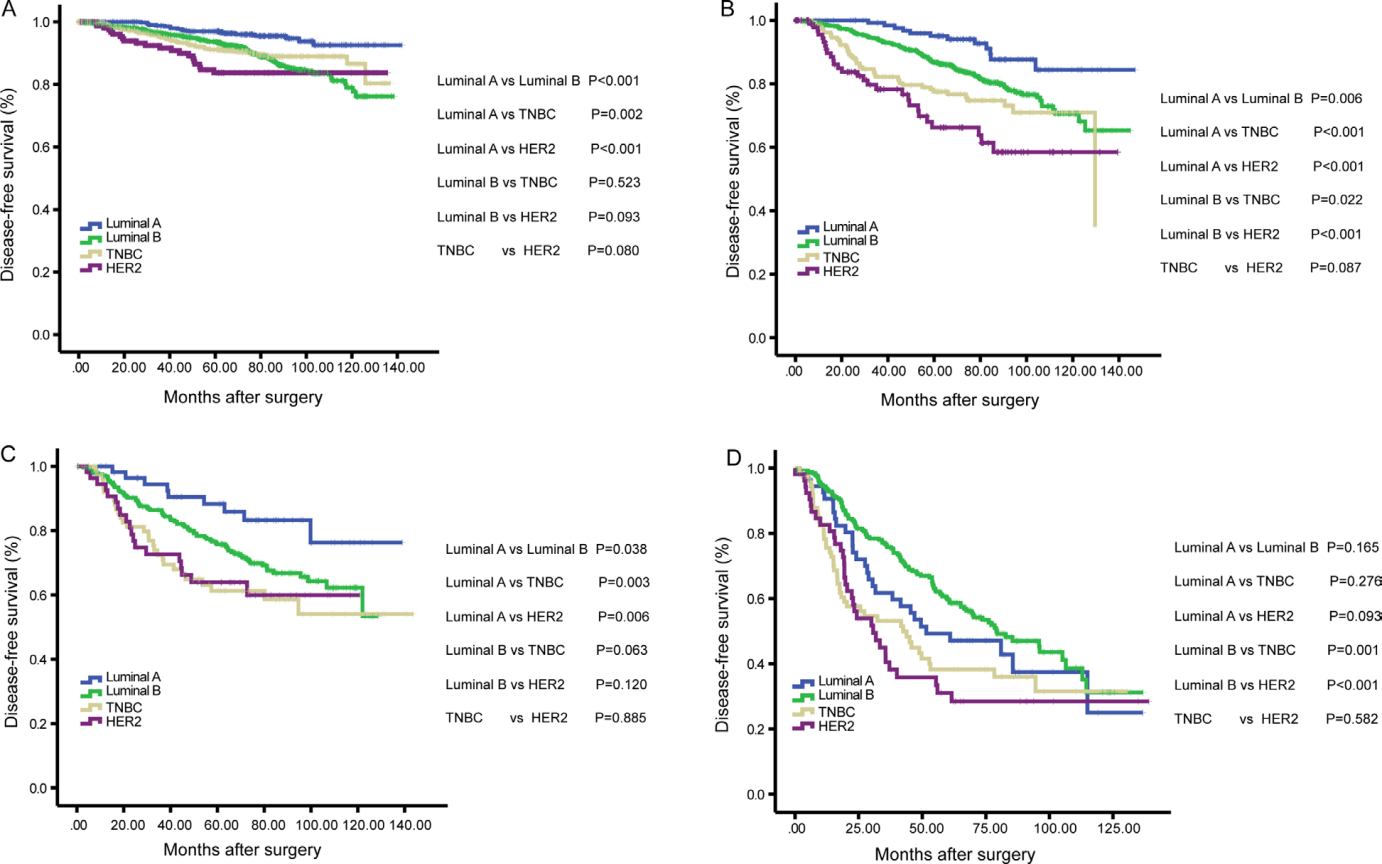
Kaplan-Meier analysis of the disease-free survival (DFS) according to the breast cancer subtypes among patients in pN0 (**A**), pN1 (**B**), pN2 (**C**) and pN3 (**D**) stage.

As shown in Figure 2A, Kaplan-Meier analysis revealed that patients in pN3 stage had an exceptionally poor prognosis: 5-year DFS rate was 93.1% in pN0 stage, 84.5% in pN1 stage, 73.5% in pN2 stage and 51.1% in pN3 stage (*P* < 0.001). In the first 5-years after surgery, Luminal A and Luminal B breast cancer patients had better DFS when compared to TNBC and HER2 breast cancer patients, however, as to 10-year DFS, the survival curves showed that there was no significant survival difference among Luminal B, TNBC and HER2 patients (Figure 2B). When the analysis was stratified by pN stage, as shown in Figure 3, we found that the patients with higher pN stage disease had poor outcome than the patients with lower pN stage disease in every breast cancer subtype in general. However, there was no significant survival difference in patients with pN1 and pN2 stage disease in Luminal A (*P* = 0.011) and Luminal B (*P* = 0.67). Furthermore, after the analysis was stratified by breast cancer subtype, we found that although in the same pN stage (pN0-pN2), there was significant survival difference among patients in different breast cancer subtypes, and Luminal A breast cancer patients had the best survival outcome (Figure 4A–4C). However, there were no significant survival difference between Luminal A patients and other breast cancer subtype when patients in pN3 stage (Figure 4D).

## DISCUSSION

Lymph nodes metastases and breast cancer subtype are both well-recognized prognostic indicators, however, whether there is an association between nodal metastases and breast cancer subtypes is still controversial and the prognostic value of nodal metastases in different breast cancer subtypes is still remains unclear. To our knowledge, this is the first study to investigate the association between nodal metastases and breast cancer subtypes in a large sample size and analyze the prognostic value of nodal metastases in different breast cancer subtypes. In our present study, we conducted a retrospective analysis of 4, 262 patients with a diagnosis of invasive ductal carcinoma of the breast.

Whether there was a significant association between breast cancer subtype and lymph node status was still unclear. Several studies revealed that there was a statistically significant association between breast cancer subtype and lymph node metastases [12–16], whereas another study revealed that there was no association between breast cancer subtype and lymph node metastases [17]. Our study showed that Luminal A and TNBC breast cancer subtypes may predict a lower risk of lymph node metastases when compared to luminal B and HER2 cancers, which was consistent with Gangi's study and Mazouni's study [16, 18]. However, other studies have suggested that there was a more likelihood of nodal metastases in TNBC breast cancer patients [13, 15]. The reasons for the discrepancy may include: (1) the sample size of previous study were relatively small; (2) the subtype definitions were different from this present study. Therefore, further studies are needed to confirm these results. In addition, as shown in Figure 1, our study revealed that both Luminal A and TNBC breast cancer subtypes were less likely to have more than nine lymph nodes metastases (in pN3 stage) when compared with Luminal B and HER2 overexpression breast cancer subtypes. In consistent with Slamon's study [19], our present study showed that HER2 overexpression is not only associated with a greater number of involved nodes, but also associated with a poor histologic grade (*P* < 0.001).

Besides, our study found higher incidence of TNBC in younger (*P* = 0.048), pre-menopausal women (*P* = 0.023), which corroborates findings in other studies [9, 21]. In consistent with Bauer's study, we found TNBC patients are more likely to present higher pT stage diseases [21].

Previously studies have demonstrated that lymph node metastases or breast cancer subtype were independent well-recognized prognostic indicators [2–8]. In accordance with these studies, we found that patients with higher pN stage disease showed a worse DFS than those patients with a lower pN stage disease. As shown in Figure 2A, Kaplan-Meier analysis revealed that patients in pN3 stage had an exceptionally poor prognosis: 5-year DFS rate was 93.1% in pN0 stage, 84.5% in pN1 stage, 73.5% in pN2 stage and 51.1% in pN3 stage (*P* < 0.001). As shown in Figure 2B, the 5-year DFS was 92% for Luminal A, 85.6% for Luminal B, and 80.0% and 68.3% for TNBC and HER2-overexpression breast cancer, respectively. However, as to 10-year DFS, there seemed no survival difference among Luminal B, TNBC and HER2-overexpression breast cancer patients. Saphner et al [22] wisely stated that “perhaps the long-term recurrence rate for ER-positive and ER-negative patients will be the same but with the ER-negative recurrences occurring more frequently in early follow-up and the ER-positive recurrences occurring in late follow-up.” In consistent with this statement, our present study demonstrated that the 10-year DFS for Luminal B, TNBC and HER2 breast cancers were similar, TNBC and HER2 breast cancer recurrences occurring more frequently in early follow-up and Luminal B recurrences occurred both in early and late follow-up. So we should pay much attention on Luminal B breast cancer patients because they have continuously higher hazard ratio of recurrence over time compared with Luminal A breast cancer patients.

It is important to note that although TNBC is more aggressive, it does not metastasize more frequently to the axilla and it is not associated more frequently with a pN3 stage disease. Similar to our study, Crabb et al. reported that the TNBC tumors, despite their poor prognosis, are associated with a lower incidence of axillary nodal involvement than other subtypes [12]. Whether pN stage play an important role in affecting the survival outcome of patients with different breast cancer subtypes is still unknown. To answer this question, we next investigate the prognostic value of nodal metastases in different breast cancer subtype patients. When the analysis was stratified by pN stage, as shown in Figure 3A–3D, we found that DFS were worse in patients with higher pN stage disease than patients with lower pN stage disease in every breast cancer subtype in general. To our interest, we found that Luminal A patients had an acceptable survival outcome even with pN2 stage disease, however, the survival was significantly decreased in Luminal patients with pN3 stage. The 5-year DFS of Luminal A patients was 96.7% in pN0 stage, 95.1% in pN1 stage, 88.3% in pN2 stage and 47.2% in pN3 stage (*P* < 0.001, pN3 vs. pN0, pN1, pN2). Since the nodal status is well -recognized as one of the strongest prognostic factors in breast cancer, it was expected to show its prognostic value also in TNBC patients. As shown in Figure 3C, the patients with higher pN stage disease had a worse DFS comparing to patients with lower pN stage disease (*P* < 0.001, pN0 vs. pN1, pN2, pN3; *P* < 0.001, pN1 vs. pN2, pN3; *P* = 0.002, pN2 vs. pN3). These results are in line with some previous studies [9, 23–25]. However, some other studies did not confirm the prognostic value of the nodal status in TNBC patients [2, 26]. Similarly, the pN stage was still discriminative to reflect the survival outcome in Luminal B, and HER2-overexpression tumors.

When the analysis was stratified by breast cancer subtypes, as shown in Figure 4A–4D, we found that Luminal A was statistically significantly associated with better DFS when compared with the other subtypes, and TNBC and HER2 subtypes had worse survival outcome over the first five years after surgery in pN0-pN2 stage (all *P* < 0.05). However, no significant survival benefit was observed in Luminal A patients when compared with TNBC and HER2 subtypes in pN3 stage (*P* = 0.276 and *P* = 0.093, respectively). That is to say, although breast cancer subtypes exert great influence on the survival outcome of breast cancer patients in pN0-pN2 stage diseases, it was not discriminative to reflect the survival outcome when in pN3 stage. So, breast cancer subtype still cannot replace axillary lymph nodes as the most important prognostic factor in breast cancer patients.

The current study may have many limitations. Firstly, there may have been a lack of uniformity because the surgery was performed by different surgeons. Secondly, our study included the patients admitted to our hospital from 2003 to 2010 and some of the adjuvant therapies administered in this present study do not represent current clinical practice (eg, most patients with ER/PgR-positive disease received tamoxifen-based endocrine therapy, and most of patients with HER2-positive disease received no trastuzumab therapy). Therefore, a large, prospective, and randomized controlled multi-center study will be important to validate our findings in this study.

## CONCLUSIONS

The results of our present study showed that there was a statistically significant association between breast cancer subtype and lymph node metastases. Furthermore, Luminal A and TNBC breast cancer subtypes may predict a lower risk of lymph node metastases when compared to luminal B and HER2 cancers. The lymph node status and molecular subtype played important roles in the outcome of breast cancer patients and they still seemed to be the most important indicators of prognosis.

## MATERIALS AND METHODS

### Patients

We conducted a retrospective analysis of 4, 262 patients with a diagnosis of invasive ductal carcinoma of the breast who underwent a surgery in the Tianjin Medical University Cancer Institute and Hospital (Tianjin, China) from January 2003 and December 2010. All patients received modified radical mastectomies. None of the patients received irradiation or chemotherapy before the surgery. Patients with a prior history of cancer or bilateral tumors were excluded. A mean age of patients was 50 (range 19–85) years old. Patients were followed up until 30 December 2015, the mean follow-up was 67 (range 3–147) months, and disease-free survival data was available in all patients examined. The patient clinicopathlogical characteristics were obtained retrospectively from the medical records and evaluated as prognostic factors. Age at diagnosis, pT stage, pN stage, ER, PgR, HER-2, P53 and Ki67 status labeling index were obtained from the case history. The expression status of ER, PgR, P53 and Ki67 labeling index was determined using the IHC method. ER- or PgR-negative status was defined as less than 1% immunoreactive cells, in accordance with recent guidelines [27]. HER-2-positive was defined as a tumor with 3-positive on IHC exam, or amplification on fluorescence in hybridization (FISH) test. After surgery, 3, 643 patients received adjuvant chemotherapy and 798 patients received adjuvant radiotherapy. All the patients with ER+/PR+ tumor received hormonal therapy at least for 5 years. Informed consent was obtained from all the patients above and research protocol for this study was approved by the Ethics Committees at the Tianjin Medical University Cancer Institute and Hospital.

### Subtype definitions

Tumors were classified into four subtypes by using IHC surrogates. A patient's tumor was considered TNBC if ER and PgR were both absent and HER2 status was negative; HER2-overexpression if ER and PgR were both absent and HER2 was positive; luminal A if ER- and/or PgR-positive, HER2-negative, and Ki-67 less than 14%; or luminal B if ER- and/or PgR-positive, HER2-negative, and Ki-67 ≥ 14% or if ER- and/or PgR-positive and HER-2 was positive. The Ki-67 labeling index cutoff of 14% was selected to recapitulate luminal A and luminal B intrinsic subtypes [11].

### Statistical analysis

DFS was defined as the duration of time between the date of the first surgery and the date of first local recurrence or distant metastasis. The χ2 test was used to analyze the correlation between breast cancer subtypes and clinical parameters. Survival curves were estimated using the Kaplan-Meier method and compared to the log-rank test. Survival analysis performed by the Kaplan–Meier method and the log-rank test was used for single-factor analysis. For multivariate analysis, Cox proportional hazard test was applied. We determined the variables for multivariate analysis that showed a statistical significance in univariate analysis for DFS. Multivariate survival analysis was performed using a stepwise forward procedure to derive a final model of the variables that had a significant independent relationship with DFS. *P* values < 0.05 were considered statistically significant. All tests were two-sided. All statistical analyses were carried out using the statistical package SPSS for windows 18.0 (Chicago, USA).

### Follow up

For all patients, follow-up started from the date of operation. They were followed up in our outpatient department every 6 months for the first 3 years after the surgery, then annually. Physical examination, ultrasound and chest X-ray were performed to observe regional recurrence or distant metastasis during the follow-up. The last follow up was until 30 December 2015, the mean follow-up was 67 (range 3–147) months, and disease-free survival data was obtained from medical records or by telephone calls or letter communication.

## SUPPLEMENTARY TABLES


