# Supplementary material for: The prognostic value of node status in different breast cancer subtypes
Source: Oncotarget. 2016 Dec 15;8(3):4563–71. doi: 10.18632/oncotarget.13943 (PMC5354854; doi:10.18632/oncotarget.13943)
Supplement: Supplementary file 1 [file oncotarget-08-4563-s001.pdf]

The prognostic value of node status in different breast cancer subtypes

Supplementary Materials

Supplementary Table S1A: Clinicopathological features and treatment modalities at presentation by breast cancer subtypes

Characteristic	Luminal A	Luminal B	Lum HER-2	TNBC	HER-2	*P* value
**pN stage**						**< 0.001**
N0	473	948	202	474	168	
N1	146	499	115	195	101	
N2	60	239	54	79	55	
N3	56	201	66	77	54	

TNBC. triple-negative, Lum HER-2. Luminal HER-2, HER-2. HER-2 overexpression
P value < 0.005 was considered to be significant, and significant *P* value was in bold font.

Supplementary Table S1B: Clinicopathological features and treatment modalities at presentation by breast cancer subtypes

Characteristic	Luminal A	Luminal B	Lum HER-2	TNBC	HER-2	*P* value
**pN stage**						**< 0.001**
N0	473	948	202	474	168	
N1–3	262	939	235	351	210	

TNBC. triple-negative, Lum HER-2. Luminal HER-2, HER-2. HER-2 overexpression
P value < 0.005 was considered to be significant, and significant *P* value was in bold font.

Supplementary Table S1C: Clinicopathological features and treatment modalities at presentation by breast cancer subtypes

Characteristic	Luminal A	Luminal B	*P* value
**pN stage**			**< 0.001**
N0	473 (64.4%)	948 (50.2%)	
N1–3	262 (35.6%)	939 (49.8%)	

TNBC. triple-negative, Lum HER-2. Luminal HER-2, HER-2. HER-2 overexpression
P value < 0.005 was considered to be significant, and significant *P* value was in bold font.

Supplementary Table S1D: Clinicopathological features and treatment modalities at presentation by breast cancer subtypes

Characteristic	Luminal A	TNBC	*P* value
**pN stage**			0.006
N0	473 (64.4%)	474 (57.5%)	
N1–3	262 (35.6%)	351 (42.5%)	

TNBC. triple-negative, Lum HER-2. Luminal HER-2, HER-2. HER-2 overexpression
P value < 0.005 was considered to be significant, and significant *P* value was in bold font.

Supplementary Table S1E: Clinicopathological features and treatment modalities at presentation by breast cancer subtypes

Characteristic	Luminal A	HER-2	*P* value
**pN stage**			**< 0.001**
N0	473 (64.4%)	168 (44.4%)	
N1–3	262 (35.6%)	210 (55.6%)	

TNBC. triple-negative, Lum HER-2. Luminal HER-2, HER-2. HER-2 overexpression
P value < 0.005 was considered to be significant, and significant *P* value was in bold font.

Supplementary Table S1F: Clinicopathological features and treatment modalities at presentation by breast cancer subtypes

Characteristic	Luminal A	Lum HER-2	*P* value
**pN stage**			**< 0.001**
N0	473 (64.4%)	202 (46.2%)	
N1–3	262 (35.6%)	235 (53.8%)	

TNBC. triple-negative, Lum HER-2. Luminal HER-2, HER-2. HER-2 overexpression
P value < 0.005 was considered to be significant, and significant *P* value was in bold font.

Supplementary Table S1G: Clinicopathological features and treatment modalities at presentation by breast cancer subtypes

Characteristic	Luminal B	TNBC	*P* value
pN stage			0.001
N0	948 (50.2%)	474 (57.5%)	
N1–3	939 (49.8%)	351 (42.5%)	

TNBC. triple-negative, Lum HER-2. Luminal HER-2, HER-2. HER-2 overexpression
P value < 0.005 was considered to be significant, and significant *P* value was in bold font.

Supplementary Table S1H: Clinicopathological features and treatment modalities at presentation by breast cancer subtypes

Characteristic	Luminal B	HER-2	*P* value
**pN stage**			0.040
N0	948 (50.2%)	168 (44.4%)	
N1–3	939 (49.8%)	210 (55.6%)	

TNBC. triple-negative, Lum HER-2. Luminal HER-2, HER-2. HER-2 overexpression
P value < 0.005 was considered to be significant, and significant *P* value was in bold font.

Supplementary Table S1I: Clinicopathological features and treatment modalities at presentation by breast cancer subtypes

Characteristic	Luminal B	Lum HER-2	*P* value
**pN stage**			0.130
N0	948 (50.2%)	202 (46.2%)	
N1–3	939 (49.8%)	235 (53.8%)	

TNBC. triple-negative, Lum HER-2. Luminal HER-2, HER-2. HER-2 overexpression
P value < 0.005 was considered to be significant, and significant *P* value was in bold font.

Supplementary Table S1J: Clinicopathological features and treatment modalities at presentation by breast cancer subtypes

Characteristic	TNBC	HER-2	*P* value
**pN stage**			**< 0.001**
N0	474 (57.5%)	168 (44.4%)	
N1–3	351 (42.5%)	210 (55.6%)	

TNBC. triple-negative, Lum HER-2. Luminal HER-2, HER-2. HER-2 overexpression
P value < 0.005 was considered to be significant, and significant *P* value was in bold font.

Supplementary Table S1K: Clinicopathological features and treatment modalities at presentation by breast cancer subtypes

Characteristic	TNBC	Lum HER-2	*P* value
**pN stage**			**< 0.001**
N0	474 (57.5%)	202 (46.2%)	
N1–3	351 (42.5%)	235 (53.8%)	

TNBC. triple-negative, Lum HER-2. Luminal HER-2, HER-2. HER-2 overexpression
P value < 0.005 was considered to be significant, and significant *P* value was in bold font.

Supplementary Table S1L: Clinicopathological features and treatment modalities at presentation by breast cancer subtypes

Characteristic	HER-2	Lum HER-2	*P* value
**pN stage**			0.611
N0	168 (44.4%)	202 (46.2%)	
N1–3	210 (55.6%)	235 (53.8%)	

TNBC. triple-negative, Lum HER-2. Luminal HER-2, HER-2. HER-2 overexpression
P value < 0.005 was considered to be significant, and significant *P* value was in bold font.
